# Protocol of systematic reviews on implementation research on cardiovascular diseases, diabetes mellitus and mental ailments in India

**DOI:** 10.12688/f1000research.128549.2

**Published:** 2024-01-16

**Authors:** Hisham Moosan, Mahendra Thakor, Arun Kumar Sharma, S. S. Mohanty, Ansuman Panigrahi, Vikas Dhikav, Suresh Yadav, Ramesh Kumar Huda, Mukesh Parmar, Poonam Singh, Denny John

**Affiliations:** 1 Department of Health Research, ICMR-National Institute for Implementation Research on Non-Communicable Diseases,, Jodhpur, Rajasthan, 342005, India; 2 Professor, Faculty of Life and Allied Health Sciences, MS Ramaiah University of Applied Sciences, Bengaluru, Karnataka, 560054, India

**Keywords:** Implementation Research, Systematic review, India, Cardiovascular Diseases, Diabetes Mellitus, Mental Health Ailments, Implementation Science, Non-Communicable Diseases

## Abstract

**Introduction:**

The burden of non-communicable diseases (NCDs) is a major public health concern across the world. Various initiatives have tried to address these with varying degrees of success.

**Objective:**

The objective is to assess and collate existing evidence in implementation research done in India on three broad domains of NCDs namely, cardiovascular diseases (CVD), diabetes mellitus (DM), and mental health (MH) in India.

**Materials and methods:**

Three systematic review protocols have been drafted to explore and collate extant evidence of implementation research on cardiovascular diseases, diabetes mellitus, and mental health in India, in accordance with the PRISMA-P statement. Academic databases including PubMed, Embase and Science Direct will be searched. Search strategies will be formulated in iterative processes and in accordance with the formats that are specific to the databases that will be searched. In addition, grey literature and non-academic databases will also be explored. Data extracted from the selected studies will be analysed and a narrative summary of the selected articles, using the SWiM (Synthesis without meta-analysis) guidelines will be produced.

**Intended Outcomes:**

The outputs of these systematic reviews could help in a better understanding of implementation research gaps and also how to address them. Apart from giving insights into how healthcare initiatives for CVDs, diabetes and mental health could be implemented in a better way, the study could also advocate the need to build and consolidate capacity for implementation research in the country.

## Introduction

The burden of non-communicable diseases (NCDs) is a major public health issue plaguing India and the rest of the world. Hence, attention to prevention, control, management, and mortality reduction of these diseases is of paramount importance. ^1^^–^^3^ Ranging across the whole spectrum of primary, secondary and tertiary prevention measures, various government initiatives have tried to address these with varying degrees of success. ^4^ Considering the fact that many of the strategies may have been proven efficient in various experimental and study settings, it is imperative to explore the real-life technical and allocative effectiveness of these programs, and the possible lacunae in their implementation.

All recent disease burden estimates point toward the fact that the burden of NCDs is increasing with each passing day. Along with this, it is important to note that the prevention and control of these diseases tend to entail longer and larger social and economic commitments on the part of governments. Thus, with a view to maximizing the utility of the efforts made by the government, it would be desirable to focus on implementation research done in this arena.

India faces a significant challenge with NCDs like cardiovascular diseases, diabetes, cancer, and chronic respiratory diseases, which are now the leading causes of morbidity and mortality. This shift from infectious to chronic diseases strains the healthcare system, which is traditionally geared towards acute care. Implementation research is crucial for addressing the gap between healthcare knowledge and its application, especially in the context of NCDs in India. It involves studying and addressing the challenges of applying research findings in real-world healthcare settings. This field is particularly significant in India, where there's a high burden of NCDs, and healthcare systems face unique challenges in terms of resources, accessibility, and cultural diversity. A review of three case studies from Zambia, Zimbabwe, and Madagascar, using the Consolidated Framework for Implementation Research (CFIR), underscores the importance of factors like leadership engagement, capacity building, and cultural adaptability in ensuring the success of these public health initiatives. In LMIC (Low- and Middle-Income countries) country like India, health interventions should integrate feasible, resource-generating elements to achieve lasting and improved health outcomes. ^5^^,^^6^

Nevertheless, it would be important to understand the quantum, diversity, and quality of implementation research done in the field of NCDs in India. Being a considerably larger domain covering a wide range of diseases, it is difficult to look at implementation research on all NCDs; hence it seemed prudent to narrow down the focus of such an inquiry to three among the most prevalent and significant disease domains, from a public health point of view, viz., cardiovascular diseases, diabetes mellitus and mental health ailments. Using a systematic review approach, this study aims to examine how IR was done to advance the prevention and control of the aforesaid three NCDs in India. The main goal of this systematic review is to describe IR that has been done in the domains of these NCDs in India and how evidence from these studies could be better applied to inform policy-level impact actions on NCD interventions in India. In this paper, we are presenting the protocol of IR that was followed in conducting the systematic review of IR in three selected NCDs.

### Objectives

To synthesize evidence on implementation research related to cardiovascular diseases (CVD), diabetes mellitus (DM), and mental health (MH) conducted in India.

Research question(s)

What is the evidence on implementation research on CVD, DM, and MH in India?

## Protocol

### Methods

With an overarching objective to assess and collate existing evidence in implementation research done in India on three broad domains of NCDs namely, cardiovascular diseases (CVD), diabetes mellitus (DM), and mental health (MH), three systematic reviews of the implementation research conducted in the aforesaid three domains of NCDs will be conducted. In accordance with extant PRISMA standards, ^7^ separate protocols have been drafted for each systematic review, which are also registered on the PROSPERO database. ( **CRD42021290547** – for Cardiovascular Diseases, **CRD42021290574** – for Diabetes Mellitus and **CRD42021290583** – for Mental Ailments). All three protocols have been written in accordance with the PRISMA for systematic review protocols (PRISMA-P) statement. ^8^

### Eligibility criteria

**Table:** 

**Population**	All age groups, who were either part or target population of an implementation project/research focused on CVDs/diabetes/mental health ailments, conducted in India
**Intervention**	Any intervention with an embedded implementation research focusing on CVDs/diabetes/mental health ailments, conducted in India
**Comparator**	Wherever applicable e.g.: Comparison studies including randomised controlled trials (RCTs), non-RCTs etc.
**Outcomes**	Assessment of reporting of implementation descriptors will be done. Subsequent to study identification, they will be examined based on whether the implementation of intervention/strategy has been described, the context of the research is mentioned, changes in IR variables are measured, who the implementing agency was, whether there were any deviations from initial protocol, whether any discussion of policy/practice implications was documented, and other relevant domains.

The following study designs will be included:

• Randomised & Non-Randomised Controlled Trials
• Observational analytical study designs, such as case control & cohort studies
• Observational descriptive designs such as cross-sectional studies
• Full or partial economic evaluation studies
• Qualitative study designs including qualitative case studies, narrative studies etc.

Studies reporting implementation research not conducted in India, study designs such as case reports, case series etc, policy briefs, editorials, letters, policy/program documents without details of study design used for implementation research, studies/reports published in any Indian language that is not understood by the investigation team member(s) or will require considerable time and effort to translate, will be excluded.

Studies, fulfilling the aforementioned inclusion and exclusion criteria, conducted during the last 20 years (2001-2021) will be included for all three systematic reviews.

### Search strategy

Academic databases including PubMed, Embase and Science Direct will be searched. Search strategies will be formulated in iterative processes and in accordance with the formats that are specific to the databases that will be searched. In addition, grey literature will be searched through various sources namely websites of programs related to non-communicable diseases and that of various departments of national and state governments, private organisations, private-public partnership organisations and non-government organisations (NGOs) engaged in healthcare. Web-based academic search engines like Google Scholar and ProQuest will also be explored. A few key government portals will be explored, and relevant government agencies will be contacted to obtain documents pertaining to implementation research, as defined in the methodology of this review. The preliminary search strategies are detailed in the appendix of this article.

### Study selection

The studies will be compiled into citation managing software. After the removal of duplicates, the titles and abstracts in three domains will be screened and assessed against the inclusion and exclusion criteria by two independent reviewers in each team using Rayyan QCRI. ^9^ Titles and abstracts will then be screened and assessed against the inclusion & exclusion criteria for the review by three independent reviewers using Rayyan QCRI software. ^9^ The eligible studies after the initial screening will be retrieved in full and will be assessed in detail against the inclusion criteria by three independent reviewers. Reasons for the exclusion of full-text studies unable to meet the inclusion criteria will be recorded and reported in the final analysis. Any disagreements between the reviewers will be resolved through discussion or consultation with the designated adjudicator wherever needed. From the articles selected for the final review, data pertaining to implementation research aspects in each of the articles will be extracted and tabulated.

### Assessment of methodological quality and risk of bias

The articles will also undergo a concomitant risk of bias assessment, using the Standards for Reporting Implementation Studies (StaRI) checklist, ^10^ and a study design-specific checklist (such as STROBE, ^11^ CONSORT ^12^ etc.) depending on the study design of the paper in question. It was also decided a priori to produce a narrative summary of the selected articles, from an implementation research perspective, using the SWiM (Synthesis without meta-analysis) guidelines. ^13^

### Data extraction

All selected studies will be grouped as per their study designs. Common to all the selected studies, data will be extracted with regard to the publication date, authors, location, setting, study population, study period and sample size. In addition to these baseline attributes, data pertaining to various domains for IR shall also be recorded.

Initially, two papers will be purposively identified, and a pilot coding form will be created. This coding form will be put up for discussion within the investigation team and will be finalized for further data extraction. Apart from the above details, the coding form will also include relevant IR outcome measures like effectiveness measures (such as uptake, patient-reported outcome measures, etc.), ^14^ economic evaluation measures (such as cost, cost-effectiveness, cost utility, etc.), ^15^ feasibility measures related to aspects such as technical, operational, economic feasibilities, operational issues (barriers, facilitating factors, etc.), fidelity measures (such as adherence, quality of intervention delivery, participant responsiveness, etc.), and scalability measures in terms of cost scalability, operational scalability, etc. ^14^

### Dissemination

All three systematic reviews shall be separately documented and respective manuscripts shall be sent for publication.

### Study status

Search and wetting of the articles completed. Narrative synthesis and manuscript preparation are currently underway.

## Discussion

This study aims to gain a better understanding of the scope and magnitude of extant IR efforts in the realm of NCDs in the country. Such a body of evidence would be integral to understanding the implementation research gaps and could pave the way to explore how they could be addressed. In addition to giving insights into how the healthcare initiatives pertaining to CVDs, diabetes and mental health could be implemented in a better way, the outputs from this study could also be a document that makes a case for the need to build and consolidate capacity for implementation research in the country.

### Limitations

Restricting our search to publications from the last 20 years (2000-2020) could potentially omit relevant studies published prior to 2000. This time frame limitation may exclude foundational research or long-term studies that could offer valuable insights into our topic. Another limitation of our review is the restriction to only three databases for sourcing literature, which may lead to potential selection bias by excluding relevant studies available in other databases not included in our search, thus limiting the comprehensiveness of our review. In addition to this, This review’s language restrictions, specifically being limited to English-language sources only.

### Ethical considerations

Not applicable.

## Data Availability

No data is associated with this article. Figshare: PRISMA-P Checklist_Protocol of Systematic Reviews on Implementation Research on Cardiovascular diseases, Diabetes Mellitus and Mental Ailments in India.pdf, https://doi.org/10.6084/m9.figshare.21556692.v1. ^16^ Figshare: Appendix I – Preliminary Search Strategy - Implementation research on Cardiovascular Diseases in India, https://doi.org/10.6084/m9.figshare.21903570.v1. ^17^ Figshare: Appendix II - Preliminary Search Strategy - Implementation research on Diabetes Mellitus in India, https://doi.org/10.6084/m9.figshare.21903729.v1. ^18^ Figshare: Appendix III - Preliminary Search Strategy – Implementation research on Mental Health in India, https://doi.org/10.6084/m9.figshare.21903732.v1. ^19^ Figshare: Appendix IV – Preliminary Data Extraction Sheet, https://doi.org/10.6084/m9.figshare.21903765.v1. ^20^ Data are available under the terms of the Creative Commons Attribution 4.0 International license (CC-BY 4.0).

## References

[1] Geldsetzer P Manne-Goehler J Theilmann M : Diabetes and Hypertension in India: A Nationally Representative Study of 1.3 Million Adults. *JAMA Intern. Med.* 2018 Mar 1;178(3):363–372. 10.1001/jamainternmed.2017.8094 29379964 PMC5885928

[2] Sreeniwas Kumar A Sinha N : Cardiovascular disease in India: A 360 degree overview. *Med. J. Armed Forces India.* 2020 Jan;76(1):1–3. 10.1016/j.mjafi.2019.12.005 32020960 PMC6994761

[3] Sagar R Dandona R Gururaj G : The burden of mental disorders across the states of India: the Global Burden of Disease Study 1990–2017. *Lancet Psychiatry [Internet].* 2020 Feb;7(2):148–161. Reference Source 31879245 10.1016/S2215-0366(19)30475-4 PMC7029418

[4] Aayog HPN : National Institution for Transforming India, Government of India.[cited 2022 Jun 26]. Reference Source

[5] Arokiasamy P : India’s escalating burden of non-communicable diseases. *Lancet Glob. Health [Internet].* 2018 Dec;6(12):e1262–e1263. 10.1016/S2214-109X(18)30448-0 Reference Source 30292427

[6] Ojo T Kabasele L Boyd B : The Role of Implementation Science in Advancing Resource Generation for Health Interventions in Low- and Middle-Income Countries. *Health Serv. Insights [Internet].* 2021 Jan 15;14:117863292199965. Reference Source 10.1177/1178632921999652 PMC7970459 33795935

[7] Page MJ McKenzie JE Bossuyt PM : The PRISMA 2020 statement: an updated guideline for reporting systematic reviews. *BMJ.* 2021 Mar 29;372:n71. 10.1136/bmj.n71 33782057 PMC8005924

[8] Moher D Shamseer L Clarke M : Preferred reporting items for systematic review and meta-analysis protocols (PRISMA-P) 2015 statement. *Syst. Rev [Internet].* 2015 Dec 1;4(1):1. 10.1186/2046-4053-4-1 Reference Source 25554246 PMC4320440

[9] Ouzzani M Hammady H Fedorowicz Z : Rayyan—a web and mobile app for systematic reviews. *Syst. Rev.* 2016 [cited 2022 Oct 18];5(1):210. 10.1186/s13643-016-0384-4 27919275 PMC5139140

[10] Pinnock H Barwick M Carpenter CR : Standards for Reporting Implementation Studies (StaRI) Statement. *BMJ.* 2017 Mar 6;356:i6795. 10.1136/bmj.i6795 28264797 PMC5421438

[11] Elm E von Altman DG Egger M : The Strengthening the Reporting of Observational Studies in Epidemiology (STROBE) statement: guidelines for reporting observational studies. *J. Clin. Epidemiol.* 2008 Apr;61(4):344–349. 10.1016/j.jclinepi.2007.11.008 18313558

[12] Schulz KF Altman DG Moher D : CONSORT 2010 Statement: updated guidelines for reporting parallel group randomised trials. *BMJ.* 2010 Mar 23;340:c332. 10.1136/bmj.c332 20332509 PMC2844940

[13] Campbell M McKenzie JE Sowden A : Synthesis without meta-analysis (SWiM) in systematic reviews: reporting guideline. *BMJ.* 2020 Jan 16;368:l6890. 10.1136/bmj.l6890 31948937 PMC7190266

[14] Lewis CC Mettert KD Dorsey CN : An updated protocol for a systematic review of implementation-related measures. *Syst. Rev.* 2018 Dec;7(1):66. 10.1186/s13643-018-0728-3 29695295 PMC5918558

[15] Husereau D Drummond M Petrou S : Consolidated Health Economic Evaluation Reporting Standards (CHEERS) statement. *BMJ.* 2013 Mar 25;346(mar25 1):f1049. 10.1136/bmj.f1049 23529982

[16] Thakor M Moosan H : PRISMA-P Checklist_Protocol of Systematic Reviews on Implementation Research on Cardiovascular diseases, Diabetes Mellitus and Mental Ailments in India.pdf. figshare.Preprint.2022. 10.6084/m9.figshare.21556692.v1 PMC10904959 38434648

[17] Moosan H Thakor M : Appendix I – Preliminary Search Strategy – Implementation research on Cardiovascular Diseases in India. figshare.Preprint.2023. 10.6084/m9.figshare.21903570.v1

[18] Moosan H Thakor M : Appendix II – Preliminary Search Strategy – Implementation research on Diabetes Mellitus in India. figshare.Preprint.2023. 10.6084/m9.figshare.21903729.v1

[19] Moosan H Thakor M : Appendix III – Preliminary Search Strategy – Implementation research on Mental Health in India. figshare.Preprint.2023. 10.6084/m9.figshare.21903732.v1

[20] Moosan H Thakor M : Appendix IV – Preliminary Data Extraction Sheet. figshare.Preprint.2023. 10.6084/m9.figshare.21903765.v1

