# Supplementary material for: Protocol of systematic reviews on implementation research on cardiovascular diseases, diabetes mellitus and mental ailments in India
Source: F1000Res. 2024 Jan 16;12:100. Originally published 2023 Jan 27. [Version 2] doi: 10.12688/f1000research.128549.2 (PMC10904959; doi:10.12688/f1000research.128549.2)
Supplement: Peer review history

# Reviewer report: Reviewer response for version 2

**DOI:** 10.5256/f1000research.161334.r238297

**Published:** 2024-02-16

**Authors:** Bishal Gyawali

**Affiliations:** 1 University of Copenhagen, Copenhagen, Denmark

All of the requests for revisions have been addressed.

Is the study design appropriate for the research question?

Yes

Is the rationale for, and objectives of, the study clearly described?

Partly

Are sufficient details of the methods provided to allow replication by others?

Yes

Are the datasets clearly presented in a useable and accessible format?

Partly

Reviewer Expertise:

Non-communicable diseases in Primary Care, Implementation Science, Global Health

I confirm that I have read this submission and believe that I have an appropriate level of expertise to confirm that it is of an acceptable scientific standard.

# Reviewer report: Reviewer response for version 1

**DOI:** 10.5256/f1000research.141150.r217931

**Published:** 2023-11-27

**Authors:** Bishal Gyawali

**Affiliations:** 1 University of Copenhagen, Copenhagen, Denmark

I appreciate the opportunity to review the manuscript protocol entitled "Protocol of systematic reviews on implementation research on cardiovascular diseases (CVDs), diabetes mellitus, and mental ailments in India." The relevance of the topic cannot be overstated, considering the underutilization of implementation research in India, where exploration of strategies for integrating evidence-based practices into real-world settings is crucial. I have several suggestions to improve the manuscript.

 In the introduction, the authors rightly emphasize the significant public health challenge posed by major non-communicable diseases (NCDs), including CVDs, diabetes mellitus, and mental ailments in India, necessitating attention to prevention, control, management, and mortality reduction. However, the references cited to support these statements primarily focus on CVDs and diabetes mellitus, neglecting mental ailments. I recommend including references related to the increasing prevalence of mental health conditions in India.

 The introduction could be strengthened by reporting the burden and consequences of the escalating NCD burden in India. Additionally, it is suggested that the authors provide a brief explanation of implementation research, its significance, and why it is needed in the Indian context, with a focus on NCDs.

 The protocol lacks clarity regarding the inclusion of interventions, as the authors mention "any intervention with an embedded implementation research." For instance, interventions could be at the system, organizational, site, or patient level. I recommend the authors define these interventions clearly and provide examples in their review.

 The rationale for limiting the review to the last 20 years (2001-2021) requires clarification.

 It is not clear if there are language restrictions in the review.

 The authors stated that non-academic databases such as Google Scholar and ProQuest will be utilized, but it should be clarified that both Google Scholar and ProQuest are web-based academic search engines that catalog records of academic and grey literature. Please reconsider this statement.

 When using acronyms such as PRISMA-P, spell out the full name the first time it is used, and similarly, spell out acronyms the first time they are introduced, avoiding repetition thereafter.

 The choice of only three databases (PubMed, Embase, and Science Direct) needs justification.

 Finally, it is important to acknowledge and discuss potential limitations in the review. There might be substantial implementation research carried out but never published, potentially introducing significant selection bias to the review.

Is the study design appropriate for the research question?

Yes

Is the rationale for, and objectives of, the study clearly described?

Partly

Are sufficient details of the methods provided to allow replication by others?

Yes

Are the datasets clearly presented in a useable and accessible format?

Partly

Reviewer Expertise:

Non-communicable diseases in Primary Care, Implementation Science, Global Health, Low-and Middle-Income Countries

I confirm that I have read this submission and believe that I have an appropriate level of expertise to confirm that it is of an acceptable scientific standard, however I have significant reservations, as outlined above.

# Reviewer report: Reviewer response for version 1

**DOI:** 10.5256/f1000research.141150.r162256

**Published:** 2023-02-27

**Authors:** Zinia Nujum

**Affiliations:** 1 Department of Community Medicine, Government Medical College, Parippally, Kerala, India

**Protocol of systematic reviews on implementation research on cardiovascular diseases, diabetes mellitus and mental ailments in India**

 General comment - This will be a very useful review and will be worth publishing. Since the authors have published the search strategy also and included them as appendices, it will be useful for future references. Hence I recommend that the protocol may be taken up for publication.

 Specific comments- Here are a few suggestions, which may be considered before publication:

 Title states “Protocol for systematic reviews on “does the author intend to conduct multiple reviews? In this case, multiple protocols will be required

 CVD, Diabetes Mellitus and Mental ailments are three very broad areas, the search terms -keywords and search strategies would be different. Also, the databases that are searched could be different, for example for mental ailments - PsycINFO covers psychology and behavioral science. This database may not be useful for other areas. So I would suggest having separate protocols for the three areas.

 It would be better to mention a Protocol for systematic review- the word FOR sounds better to me than OF.

 Search strategies will be formulated in iterative processes and in accordance with the formats that are specific to the databases that will be searched- shouldn’t we fix the search strategy a priori.

 “Academic databases including PubMed, Embase, and Science Direct will be searched. “- It would be good to add a few more if possible at least Cochrane Library also can be included since the design of the studies includes trials.  Also consider some more databases like CINAHL EBSCO, Global Health Ovid, and HMIC Ovid.

 Under ethical considerations, it can also be written how the review will be beneficial 

Is the study design appropriate for the research question?

Yes

Is the rationale for, and objectives of, the study clearly described?

Yes

Are sufficient details of the methods provided to allow replication by others?

Yes

Are the datasets clearly presented in a useable and accessible format?

Not applicable

Reviewer Expertise:

Infectious Diseases Epidemiology

I confirm that I have read this submission and believe that I have an appropriate level of expertise to confirm that it is of an acceptable scientific standard.
